# A pictorial (and hopefully pedagogical) discussion on the Born–Oppenheimer approximation

**DOI:** 10.1007/s10698-025-09559-9

**Published:** 2025-11-15

**Authors:** Federica Agostini, Basile F. E. Curchod

**Affiliations:** 1 Université Paris-Saclay, CNRS, Institut de Chimie Physique UMR8000, 91405 Orsay, France; 2 Centre for Computational Chemistry, School of Chemistry, University of Bristol, Bristol, BS8 1TS UK

**Keywords:** Born-Oppenheimer approximation, Molecular dynamics, Theoretical chemistry, Nonadiabatic dynamics

## Abstract

The Born–Oppenheimer approximation is one of the central tenets of chemical dynamics and reactivity. Despite its central importance in chemistry, the Born–Oppenheimer approximation is often severely misinterpreted or misrepresented. More specifically, widespread claims about the Born–Oppenheimer approximation within the chemistry community imply that this approximation enforces the nuclei of a molecule to be (1) frozen and (2) treated as classical particles. Both claims are wrong. This article aims to discuss the derivation of the Born–Oppenheimer approximation in a pedagogical way, representing the main steps of this derivation pictorially via a model molecular system. The pictorial derivation is then connected to the formal mathematical derivation for the interested reader. We hope that this pictorial derivation can help chemists to better understand the main steps leading to this key approximation and why the two claims mentioned above are incorrect. We also believe that a detailed understanding of the Born–Oppenheimer approximation is necessary to tackle problem in chemistry where this approximation breaks down, like photochemistry for example.

## Introduction

Molecules are complex quantum-mechanical objects, resembling to a soup of electrons and nuclei with no hierarchy per se in their mutual interaction. Nonetheless, molecules are often pictured as nuclei connected together by electrons. This picture relies on the Born–Oppenheimer representation of the quantum-mechanical reality, allowing us to regard the effect of the electrons as a potential dictating the behavior of the nuclei. The Born–Oppenheimer representation is merely a mathematical expedient to introduce a hierarchy in the electron-nuclear interactions, and find its roots in the so-called Born–Oppenheimer approximation (Born and Oppenheimer 1927). In short, the Born–Oppenheimer approximation separates the motion of the electrons from that of the nuclei forming a molecule (we will explain and justify this statement below).

A large number of excellent reviews, articles, and monographs have discussed in detail the mathematical aspects of the Born–Oppenheimer approximation and what it implies for a molecule—the interested reader can refer to Tully (2000), Marx and Hutter (2009), Heller (2018), Agostini and Curchod (2019), for example. Despite the vast amount of literature on the Born–Oppenheimer approximation and it being the tenet of modern chemistry, it is surprising to realize how misunderstood this approximation is by chemists (and physicists). Two main misinterpretations of the Born–Oppenheimer approximations can often be heard or read: (1) The Born–Oppenheimer approximation implies that the nuclei of a molecule are frozen, i.e., fixed at one given molecular geometry (or, *clamped*), and (2) the Born–Oppenheimer approximation considers that the nuclei are treated classically (in contrast to quantum mechanically). Spoiler alert: both statements are *incorrect*!

These misconceptions could have emerged from the challenge for chemists to approach the mathematical aspects of the Born–Oppenheimer approximation, especially when this approximation is only explained via the limit $m/M\rightarrow 0$ of the time-(in)dependent molecular Schrödinger equation, where *m* and *M* are the electronic and nuclear masses, respectively. Hence, we propose in the following a ’pictorial derivation’ of the Born–Oppenheimer approximation, where a simple molecular system is used to illustrate the key quantities and different steps leading to the (modern) Born–Oppenheimer approximation. To reinforce the pedagogical content of this work, we then propose, for the interested reader, a parallel mathematical derivation matching the steps of the pictorial one. Our pictorial derivation focuses on the properties of the molecular wavefunction in a dynamical context, such that the Born–Oppenheimer approximation relies on considerations based on time scales and energy differences, without the need to invoke the $m/M\rightarrow 0$ limit.

This article aims to (i) provide insight and a better intuition for what the Born–Oppenheimer approximation means for a molecular system, (ii) discuss when this approximation is expected to work and when it fails, and (iii) debunk a few myths and shortcuts often wrongly associated to this approximation. Section 2 introduces the main ’problem’ addressed by the this approximation – treating the evolution of a molecular state (or molecular wavefunction) purely with quantum mechanics – and discusses the steps leading to the Born–Oppenheimer approximation by using a simple molecular system. These steps closely follow the usual derivation of the Born–Oppenheimer approximation that can be found in several monographs, but avoid the use of any equations. The more mathematically-oriented readers will then find the full derivation in Sect. 3 and can connect it to our pictorial discussion. We stress at this point that the purpose of this article is purely pedagogical and targets a better understanding of the Born–Oppenheimer approximation for chemists. Our work does not offer a discussion on the more mathematical questions related to the Born–Oppenheimer (and Born–Huang) representation – several recent works addressed these in details, e.g., Huggett et al. (2024) or Scerri (2025).

## A pictorial derivation of the Born–Oppenheimer approximation

**Step 1: Definition of the molecular wavefunction and molecular Schrödinger equation** Let us first ask ourselves: what is the general context in which the Born–Oppenheimer approximation is found? The answer is quantum mechanics, and more specifically how to describe a molecule within the context of quantum mechanics. In quantum mechanics, the *state* of a system is described by a wavefunction, which is a complex function that depends on all the coordinates of the system of interest. The state of the system is not the system per se, but it is a set of variables that defines uniquely the observed behavior of the system. In classical mechanics, the state of a system is entirely defined by its position and momentum at a given time (its so-called phase-space representation). In quantum mechanics, the state of the system is defined by an abstract, multidimensional quantity called the wavefunction, which provides information about experimental observables when questioned by mathematical operators, such as the position and the momentum operators as well as the spin, the energy and so on. In other words, the wavefunction of a (pure) system encodes all the information about this system, and operators question the wavefunction to output a given observable related to the property of the system.

In the context of chemistry, we will focus on the ’molecular wavefunction’, that is, a wavefunction that depends on the position of all the electrons and nuclei of a molecule. In addition, quantum mechanics requires another key element to specify the system of interest, the so-called Hamiltonian, which, in the present case, groups all the possible contributions to the total energy of our molecule: the kinetic energy of all its nuclei, the kinetic energy of all its electrons, as well as all the electrostatic interactions between electrons, between nuclei, and between electrons and nuclei.

The molecular wavefunction and the Hamiltonian are connected by the molecular Schrödinger equation. In its stationary version, the molecular Schrödinger equation shows that, for a proper wavefunction of our system (a so-called eigenfunction), the Hamiltonian operator applied to the the molecular wavefunction returns the very same molecular wavefunction multiplied by a constant: this constant describes the molecular energy of our system. The time-dependent molecular Schrödinger equation, instead, indicates how a given molecular wavefunction, i.e., the initial condition, evolves in time under the influence of the molecular Hamiltonian, and, as such, predicts how the state of our molecule changes over time. In both its stationary and time-dependent case, the molecular Schrödinger equation is excruciatingly difficult – if not impossible – to solve exactly due to its inherent mathematical complexity. One of the key motivation for the Born–Oppenheimer approximation is to split the molecular Schrödinger equation into two (coupled) equations, an electronic and a nuclear equation, simplifying its resolution (when the conditions for its validity are met). In the following, we will focus on the time-dependent molecular Schrödinger equation as it offers a clearer depiction of the role of the Born–Oppenheimer approximation.

**Fig. 1:**
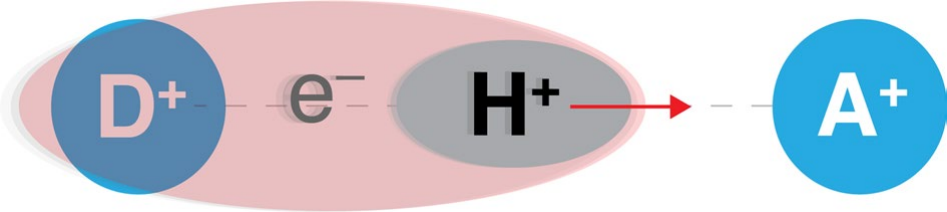
Schematic depiction of a simple molecular system: the motion of a proton ($\textrm{H}^{+}$) and an electron ($\textrm{e}^{-}$) along a one-dimensional coordinate (dashed grey line) connecting a fixed donor (D$^+$) and acceptor (A$^+$). The donor and acceptor (blue circles) are both charged positively.

**Step 2: Introducing a molecular model system for this pictorial derivation** The previous paragraph set the scene for the pictorial derivation of the Schrödinger equation by introducing its key actors: the molecular wavefunction and the molecular Hamiltonian. Let us now define a very simple ’molecular’ system to motivate our derivation: the transfer of a hydrogen atom along a one-dimensional line connecting a donor (D) to a acceptor (A), both fixed in space. This model can be used in different regimes, depicting either a simple hydrogen transfer or a proton-coupled electron transfer ($\textrm{H}^{+} +\textrm{e}^{-}$) between a donor and an acceptor. We will consider the latter in the following, meaning that our model represents the following situation (further depicted in Fig. 1):

$$\begin{aligned} \textrm{D}-\textrm{H} + {\textrm{A}^{+}} \longrightarrow [{\textrm{D}^{+}} +\textrm{H}^{+} +\textrm{e}^{-} + {\textrm{A}^{+}}] \longrightarrow {\textrm{D}^{+}} + \textrm{H}-{{\textrm{A}}} \end{aligned}$$

The previous scheme highlights that the fixed donor and acceptor centres are positively charged. While simple, this model has been successfully used in the past to mimic charge transfer and different regimes of proton-coupled electron transfer in biological and chemical processes. (Shin and Metiu 1995, Fang and Hammes-Schiffer 1997) This model possesses only a single degree of freedom for both the electron and the proton, as both particles are forced to evolve solely along the one-dimensional coordinate, or bonding axis, connecting the donor to the acceptor. As a result, the molecular wavefunction for this system has two dimensions: the electronic position and the proton position (along the same one-dimensional coordinate, dashed grey line in Fig. 1). The molecular Hamiltonian is composed of the kinetic energy for the electron and the proton, their electrostatic interaction, as well as the electrostatic interaction of the proton and the electron with the positive charge of both the donor and acceptor.

**Fig. 2:**
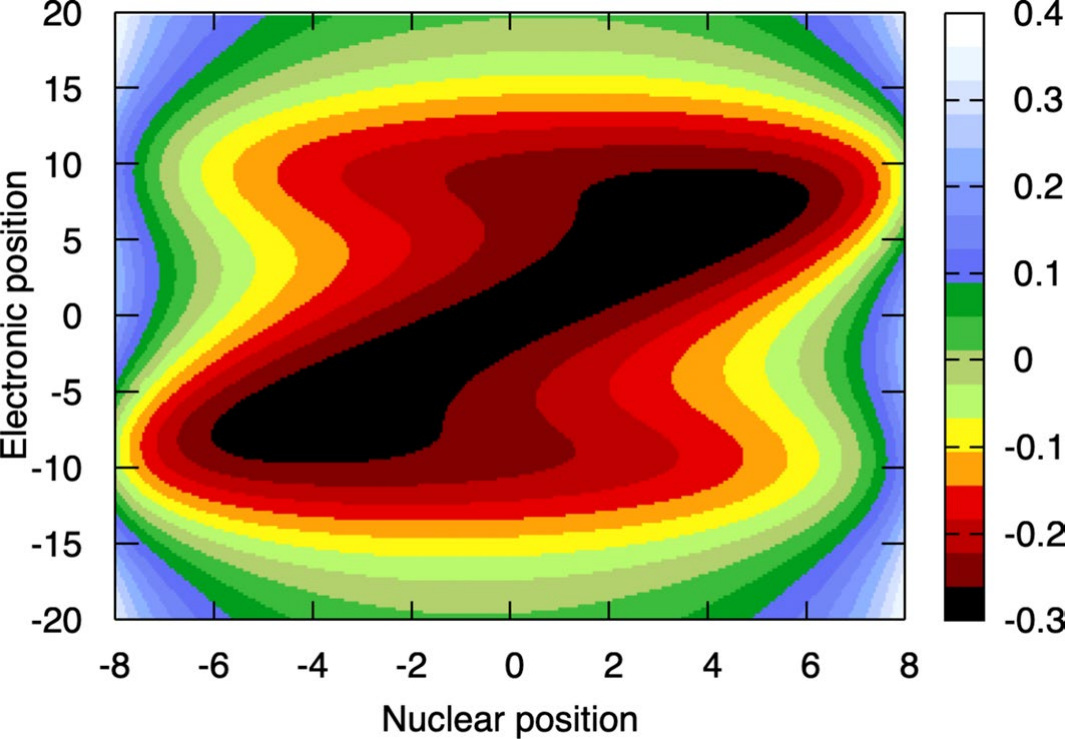
Electrostatic contributions to the molecular Hamiltonian for the simple molecule depicted in Fig. 1. The abscissa indicates the coordinate of the nucleus (the proton) and the ordinate gives the position of the electron. Note the different scale for the axis of the nuclear and electronic position. The (fixed and positively charged) donor and acceptor are located at -10 and +10, respectively. Atomic units are used throughout.

**Step 3: The electrostatic potential in the molecular Hamiltonian** Let us first investigate how the electrostatic contributions present in the molecular Hamiltonian look like for the simple molecular system. Figure 2 provides a colormap plot depicting the total electrostatic potential along its two dimensions, the position of the proton $\textrm{H}^{+}$ (abscissa, nuclear position, *R*) and the position of the electron $\textrm{e}^{-}$ (ordinate, electronic position, *r*). We should note here the different scales for the axis of the nuclear and electronic position, and that the donor and the acceptor are located at -10 and +10, respectively (atomic units are used throughout this text). This figure highlights the overall electrostatic behavior of the system, showing the correlation between the nuclear and electronic position. For example, if the proton is closer to the positive charge of the donor (negative value for the nuclear position in Fig. 2), the overall electrostatic potential will have a negative energy when the electron sits between these two charges (i.e., at an electronic position *r* between -5 and -10). Bringing the nuclear position closer to the donor or the acceptor, i.e., $R< -8$ or $R>8$, leads to a strong electrostatic repulsion between the two positive charges and a large positive electrostatic potential.

**Fig. 3:**
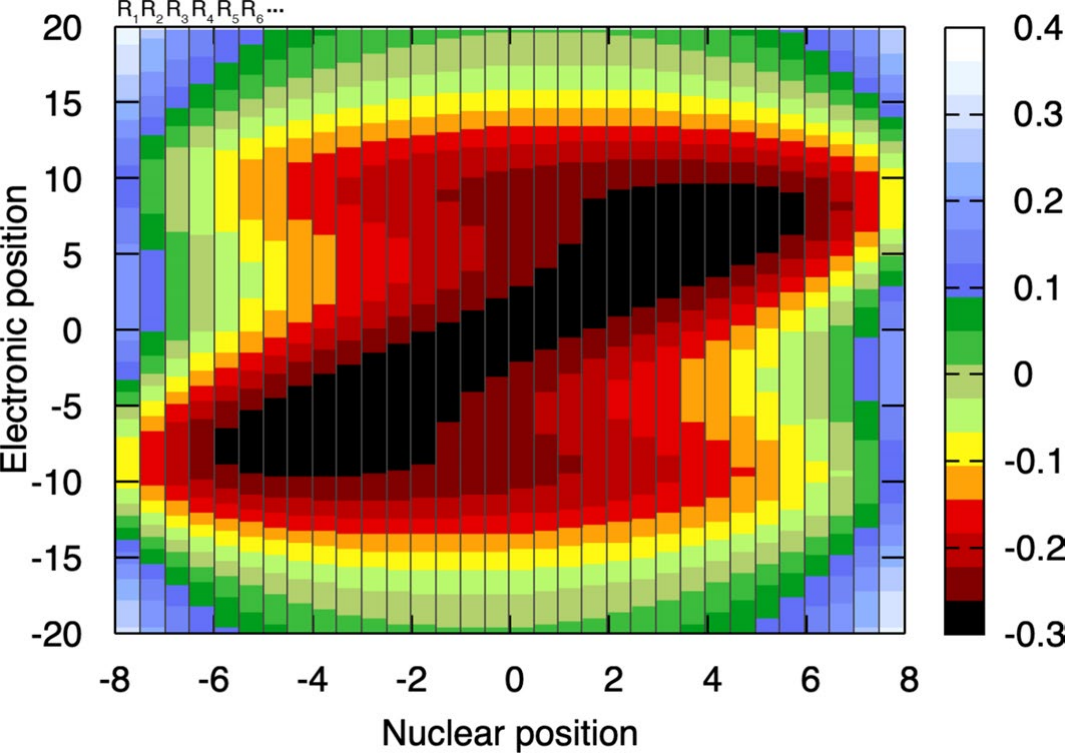
Same representation of the overall electrostatic potential as depicted in Fig. 2, but discretized along the nuclear coordinates. Each slice of nuclear coordinate is delimited by vertical gray lines and labeled $R_1$, $R_2$, $R_3$, etc. (see top of the figure).

**Step 4: Discretizing the electronic Hamiltonian along the nuclear coordinates** As stated above, the complexity of the molecular Schrödinger equation makes it impossible to solve for anything but the simplest molecules. Born and Huang suggested in 1954 a procedure to rewrite the molecular Schrödinger equation in a formalism still exact but simpler then to approximate. The idea is to *discretize* the molecular problem along the nuclear coordinates. What do we mean by that? Simply that instead of tackling the full molecular problem with all the nuclear and electronic degrees of freedom at once, we will split the electronic and the nuclear variables by slicing the total electrostatic potential along the nuclear coordinate to define a very fine (if possible, infinitely fine) grid of nuclear positions. Figure 3 schematically depicts this discretization, showing the total electrostatic potential for various discretized nuclear positions, labeled $R_1$, $R_2$, etc. (top of Fig. 3). We note that the slices here have on purpose a finite width for clarity – in principle, each slice should be infinitely narrow along each nuclear coordinate. The strategy then consists in taking a given (infinitely narrow) slice of the electrostatic potential, say $R_1$ here, and by adding to it the kinetic energy operator for the electrons, we obtain a sort of electronic Hamiltonian for this specific set of nuclear coordinates. Doing that for all slices, we can define an electronic Hamiltonian (kinetic energy of the electrons + all the electrostatic contributions for a given nuclear slice) over the full range of possible nuclear configurations.^1^

**Fig. 4:**
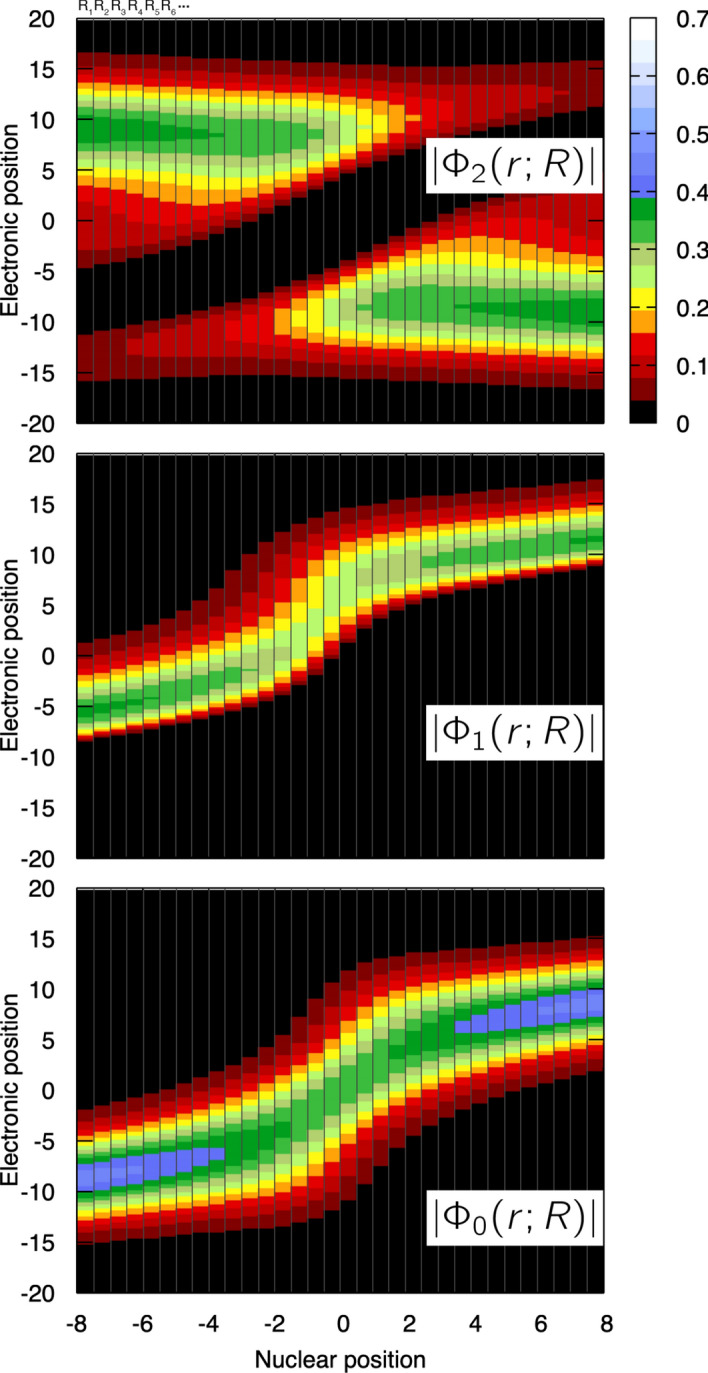
Electronic wavefunctions for the three lowest electronic states, $\Phi _0(r;R)$, $\Phi _1(r;R)$, and $\Phi _2(r;R)$. Each slice of the electronic wavefunctions (delimited by vertical grey lines) represents a solution of the electronic stationary Schrödinger equation for a given nuclear configuration, $R_1$, $R_2$, $R_3$, etc. (see top of the figure). The modulus of each wavefunction is depicted for clarity.

**Step 5: Solving the electronic Schrödinger equation for all nuclear coordinates to obtain electronic wavefunctions** How did the procedure described above help at all in our quest to solve the molecular Schrödinger equation? Defining an electronic Hamiltonian for a given set of nuclear coordinates, $R_1$ to keep our example, allows us then to solve an electronic Schödinger equation to obtain, for this specific nuclear configuration, the so-called electronic wavefunctions and electronic energies for any possible arrangements of the electron, also called electronic states, around this specific nuclear configuration (or nuclear geometry). A depiction of the (moduli of the) electronic wavefunctions for each nuclear slice of our model system is given in Fig. 4 (note that the modulus square of the electronic wavefunction represents the electronic probability density distribution). Figure 4 provides the (moduli of the) electronic wavefunctions for the ground electronic state $\Phi _0(r;R)$ – the lowest energy arrangement of the electron in our simple system – as well as the electronic wavefunction for the first two lowest excited electronic states $\Phi _1(r;R)$ and $\Phi _2(r;R)$ – we note here that there are an infinite number of electronic states. Focusing on the nuclear slice $R_1$, we can see how the ground electronic wavefunction, $\Phi _0(r;R_1)$, spreads in space, by consulting, for this slice, the y-axis of the electronic position. In this specific case, we observe that most of the electronic wavefunction spreads between an electronic coordinate of ${-12}$ and ${-5}$. This observation makes sense, considering that the proton (our nucleus) is at position R$_1\sim -7.5$, meaning that the electron benefits from the attractive coulombic interaction with the proton and the positively-charged donor located at ${-10}$. At a nuclear position closer to $R=0$, the electronic wavefunction is centered around zero too, following the position of the proton. Focusing now on the third electronic state, $\Phi _2$, at $R=0$, we can see that there is no amplitude for the electronic wavefunction in the region $r=0$, but instead, we see some amplitude between $-13$ and $-5$ and ${5}$ to ${13}$. In this excited electronic state, the electron is delocalized next to the donor and the acceptor. In summary for this paragraph, discretizing the electronic Hamiltonian along the nuclear coordinates allows us to solve, for each slice (nuclear configuration), an electronic stationary Schrödinger equation whose eigenfunctions represent the electronic wavefunctions for all electronic states at this molecular geometry. In the limit of an infinitely small discretization, we would recover the full depiction of the electronic wavefunctions for any nuclear coordinates.

**Fig. 5:**
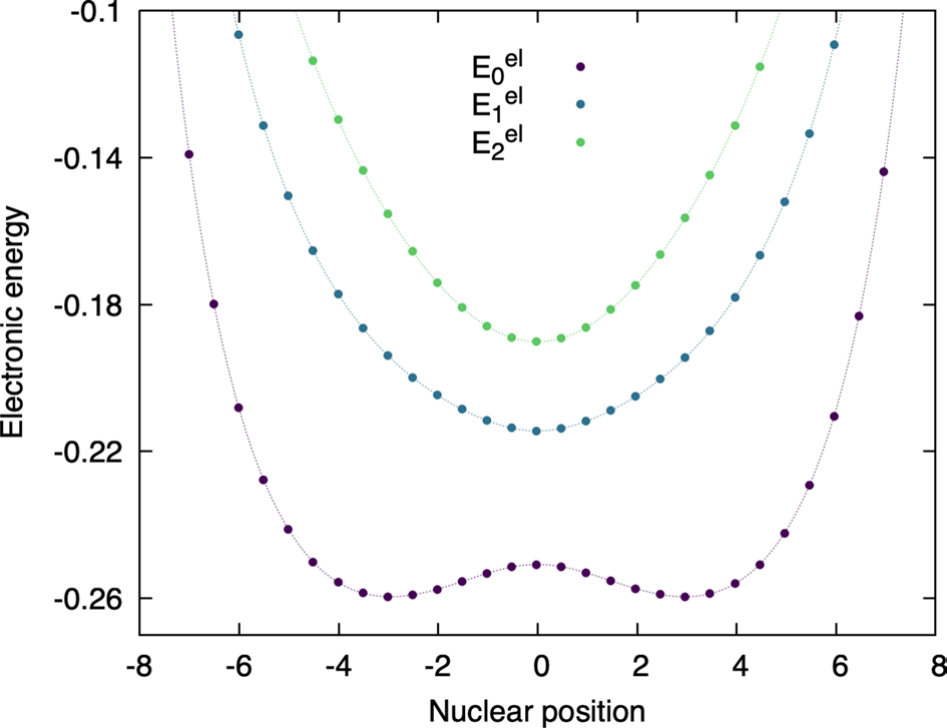
Potential energy curves (lines) emanating from plotting the eigenvalues of the electronic Schrödinger equations (dots), or electronic energies ($E_0^{\text {el}}$, $E_1^{\text {el}}$, $E_2^{\text {el}}$, etc) for each nuclear position of our simple molecular system. Different colors highlight the energies of the three lowest electronic states depicted here.

**Step 6: The eigenvalues of the electronic Hamiltonian form the potential energy surfaces felt by the nuclei** How about the eigenvalues of the electronic Schrödinger equation discussed above? These eigenvalues represent the electronic energies of the molecular system for each nuclear configuration. For example, at nuclear position $R_1$, the ground electronic state has an electronic energy $E_0^{\text {el}}(R_1)$, the first excited electronic state is $E_1^{\text {el}}(R_1)$), the second excited electronic state is $E_2^{\text {el}}(R_1)$, etc. Importantly, the electronic energies are a function of the nuclear configuration (or coordinate). Plotting the electronic energies as a function of the nuclear position gives rise to the concept of ’potential energy surfaces’, pivotal in chemists’ understanding of (photo)chemical reactivity. Figure 5 depicts the electronic energy of each electronic state and for all slices of nuclear position discussed above (filled circles). In the limit of an infinitely small discretization, we recover the potential energy surfaces (lines). This representation makes explicit the potential energy that the proton of our model (our nucleus) would feel in the ground electronic state, with a minima of energy at around $R=-3$ and $R=3$, and a transition state located at a nuclear position of zero $R=0$. This observation implies that the proton is more stable when located closer to one of the fixed charges (donor or acceptor) thanks to the electrostatic interaction created by the electron, in our model system (Fig. 1). Interestingly, the shape of the potential energy curves for the excited electronic states is dramatically different and translates to the different electronic configurations observed in Fig. 4.

**Step 7: The Born–Huang representation rewrites the molecular wavefunction as products of an electronic wavefunction times a nuclear wavefunction, summed over all electronic states** Coming back to our original goal – devising a strategy to solve the molecular Schrödinger equation—what did we gain by obtaining information about the electronic wavefunctions and electronic energies? Considering that we know the electronic wavefunctions for all electronic states and any nuclear coordinates, we can use these electronic wavefunctions as a *basis* to express the molecular wavefunction. In other words, we can express something that we do not know—the molecular wavefunction—based on a set of function—the electronic wavefunctions—that we know and can obtain by solving the electronic Schödinger equation for each nuclear configuration. This idea is at the heart of the Born–Huang representation of the molecular wavefunction. How does this expansion work, in practice? We simply write the molecular wavefunction as a sum of *products* over all the possible electronic states, where the ’products’ are composed of the electronic wavefunction for a given electronic state (we know it) times a coefficient that will indicate its importance in the molecular wavefunction (encoding the projection of the molecular wavefunction onto the corresponding electronic wavefunction). This coefficient is, in fact, also a function of the nuclear coordinates (and time for time-dependent problems) and is often coined the nuclear wavefunction for a given electronic state. Hence, the Born–Huang representation allows us to split the total molecular wavefunction into products of electronic and nuclear wavefunctions for all electronic states. We can solve first the electronic problem, determining the potential energy surfaces and electronic wavefunctions, and we can then get access to the full molecular wavefunction by solving the nuclear problem, i.e., by determining the nuclear wavefunctions, which are still unknown, on the support of the electronic quantities.

**Fig. 6:**
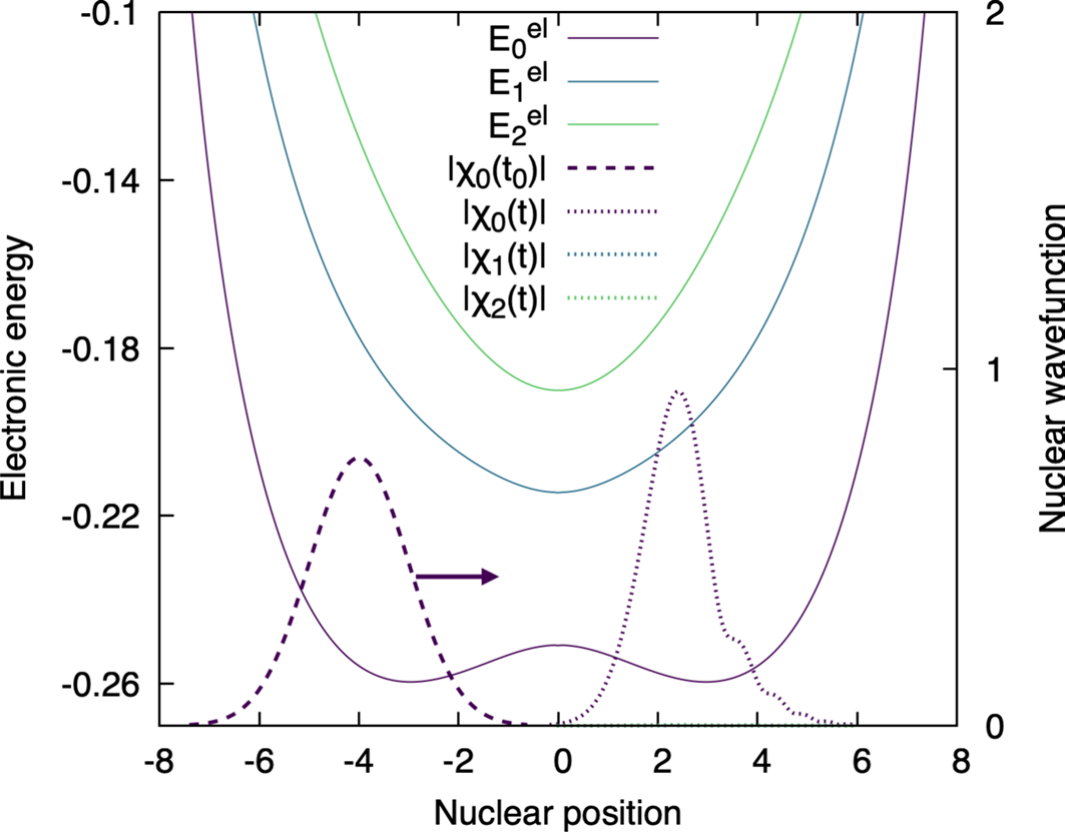
Nuclear dynamics of the model molecular system. The dashed Gaussian shape represents the initial nuclear wavefunction for the proton ($\chi _0(t_0)$), centered at $R=-4$ and launched with a positive momentum towards the positive nuclear position (arrow), in the direction of the acceptor. The system is initially in its ground electronic state, that is, only the nuclear wavefunction for the ground electronic state $\chi _0(t)$ has amplitude. The dotted curves symbolize the nuclear wavefunctions (for the three lowest electronic states, $\chi _0(t)$, $\chi _1(t)$, and $\chi _2(t)$) at time *t* of the dynamics. In this particular case, only the nuclear wavefunction for the ground electronic state, $\chi _0(t)$, has non-zero nuclear amplitude, while the two other nuclear wavefunctions are zero. The modulus of each nuclear wavefunction is depicted for clarity.

**Step 8: Within the Born–Huang representation, nuclear wavefunctions evolve on coupled potential energy surfaces** How does the Born–Huang representation look like in practice? Let us come back to our model system (Fig. 1) and study the dynamics of our proton moving from a nuclear position (around $-4$ at the beginning) close to the donor and evolving towards the acceptor (the momentum of the proton is symbolized by an arrow in Fig. 1). From a chemical perspective, we induce a proton-coupled electron transfer from the donor to the acceptor (as discussed in Section 2). Figure 6 depicts this dynamics in the Born–Huang representation: the potential energy curves for the three lowest electronic states of our model system were obtained by solving the electronic Schrödinger equation, while the dashed Gaussian shape centered at the nuclear position $R=-4$ represents the initial nuclear wavefunction associated to the ground electronic state at the beginning of the dynamics ($|\chi _0(R, t_0)|$) – note that all other nuclear contributions on any of the (excited) electronic state considered have an amplitude of zero at the beginning of the dynamics. Henceforth, even if not explicitly indicated, all the nuclear wavefunctions are to be considered functions of *R* and *t*. Note that, as the time-dependent Schrödinger equation is a partial differential equation, we have the freedom to choose this (or any other) initial condition, while the following dynamics is fully determined by the solution of the equation, as we describe henceforth. We solve a set of coupled time-dependent nuclear Schrödinger equations, one for each nuclear wavefunction on each electronic state considered, to evolve our proton on the support of the potential energy surfaces. The fact that we have to solve a set of coupled time-dependent nuclear Schrödinger equations is caused by the use of the Born–Huang representation, which depicts the total molecular wavefunction as a sum (over all electronic states) of products of nuclear and electronic wavefunctions: hence, to obtain the *exact dynamics* of the full molecular wavefunction, we must obtain, under this representation, information about each nuclear wavefunction on each electronic state.^2^ The dotted lines (on the positive side of nuclear positions) symbolize the different nuclear wavefunctions after a certain time *t* of dynamics. In other words, the proton reached a region closer to the acceptor (located at $R=+10$). We can immediately see that the ground-state nuclear wavefunction ($|\chi _0(t)|$) lost its original Gaussian shape and developed some features, mostly due to the repulsive part of the ground-state potential energy curve ($E_0^{\text {el}}(R)$) at large nuclear position values (caused by the repulsion with the positively-charged acceptor). Perhaps more interesting, though, is the fact that we cannot see any other nuclear contributions appearing in this dynamics – the amplitude of the nuclear wavefunctions at time *t* for the two other electronic states depicted, $|\chi _1(t)|$ and $|\chi _2(t)|$ are nearly zero and barely visible in our plot. In other word, the nuclear dynamics depicted here, initiated in the ground electronic state, did not lead to a significant coupling between nuclear and electronic motion that would have resulted in the nuclear population of other electronic state. In simpler words: the nuclei remain in the ground electronic state and did not visit other electronic states. The evolution of the nuclei on the support of a single potential energy surface (in a single electronic state) is often coined ’adiabatic’. This regime is achieved when the nuclear dynamics, characterized by a typical time scale $\tau _{\textrm{n}}$, is slow enough compared to the dynamics of the electrons, characterized by $\tau _{\textrm{el}}$, such that $\tau _{\textrm{el}}/\tau _{\textrm{n}}\ll 1$.

**Fig. 7:**
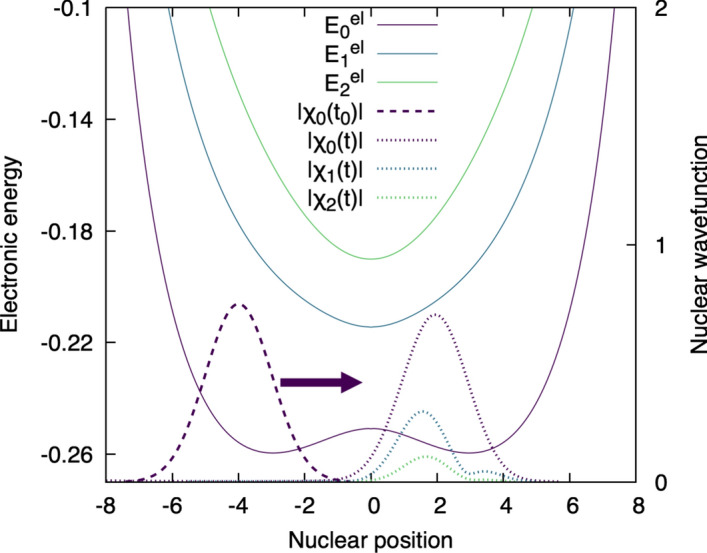
Nuclear dynamics of the model molecular system, this time with a larger initial momentum on the proton. The dashed Gaussian shape represents the initial nuclear wavefunction for the proton ($\chi _0(t_0)$), centered at $R=-4$ and launched with a positive momentum towards the positive nuclear position (arrow), in the direction of the acceptor. The system is initially in its ground electronic state, that is, only the nuclear wavefunction for the ground electronic state $\chi _0(t)$ has amplitude. The dotted curves symbolize the nuclear wavefunctions (for the three lowest electronic states, $\chi _0(t)$, $\chi _1(t)$, and $\chi _2(t)$) at time *t* of the dynamics. In this case, the ground nuclear wavefunction transferred amplitude to the other nuclear wavefunctions due to the strong coupling between electronic and nuclear motion, a process called nonadiabatic coupling or nonadiabatic transitions. The modulus of each nuclear wavefunction is depicted for clarity.

Is it always the case that a molecule evolving in a given electronic state remains in said state? No! For the very same molecular model, the picture changes dramatically if the initial momentum applied on the proton at the beginning of the dynamics is increased, i.e., the proton moves faster towards the acceptor. Figure 7 depicts this precise case, where the proton, described initially by the same $|\chi _0(t_0)|$ as above, is launched towards the acceptor with a larger initial momentum. In this particular situation, the enhanced coupling between electronic and nuclear motion leads to transfer of nuclear amplitude from the ground-state nuclear wavefunction ($\chi _0(t)$) to the first and second excited electronic states, leading to sizeable nuclear wavefunctions $\chi _1(t)$ and $\chi _2(t)$ in these electronic states – in other words, the molecule does not remain in its electronic ground state, but is now in a superposition of electronic states. Hence, the Born–Huang representation accurately depicts the coupled motion of electrons and nuclei in a molecular system, the so-called ’nonadiabatic coupling’, which can result in a transfer of nuclear amplitude between electronic states. The strength of the nonadiabatic couplings depends on the momentum of the nuclei and the energy gap separation between the electronic states: these criteria are visible in the two examples presented above, where we observe stronger nonadiabatic effects with a larger momentum for the proton, and the nonadiabatic effects take place in the region of nuclear configuration space where the electronic energy gap between the ground and the other electronic states is smaller (around a nuclear position of $R=0$). While it is clear that nonadiabatic effects are important to accurately describe the state of our molecular system, it is important to realize at this point that there might be chemical processes (like that depicted in Fig. 6) that could be very well described by neglecting these effects and focusing only on the nuclear wavefunction of a single electronic state – this train of thought leads to the Born–Oppenheimer approximation!

**Step 9: Born–Oppenheimer approximation: the coupling between electronic and nuclear motion, responsible for the transfer of nuclear amplitude between electronic states, can be neglected in some specific cases**

**Fig. 8:**
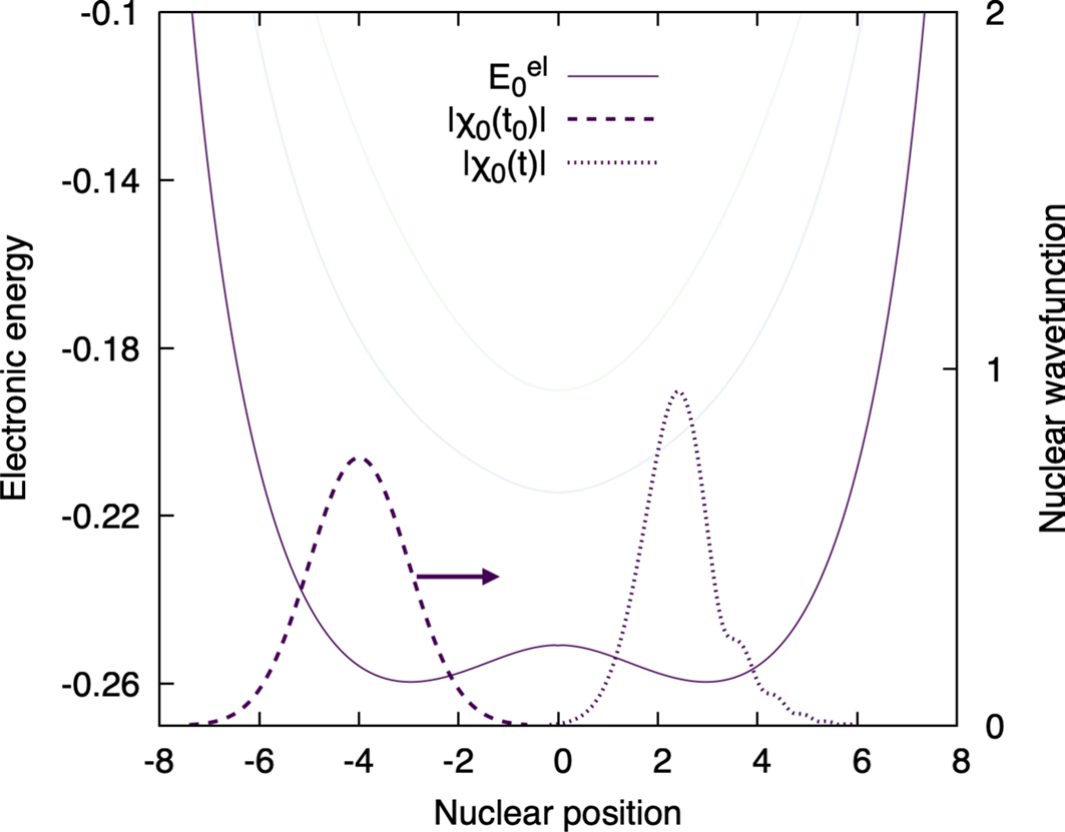
Nuclear dynamics of the model molecular system within the Born–Oppenheimer approximation. The dashed Gaussian shape represents the initial nuclear wavefunction for the proton ($\chi _0(t_0)$), centered at $R=-4$ and launched with a positive momentum towards the positive nuclear position (arrow), in the direction of the acceptor – inducing a proton-coupled electron transfer. Due to the Born–Oppenheimer approximation, the system is constrained to the ground electronic state, that is, there is only a single nuclear wavefunction $\chi _0(t)$ assigned for this state in the expression of the molecular wavefunction, evolving on the ground-state potential energy curve ($E_0^{\text {el}}$). The pale curves shows, for memory, the location of the other electronic states that are no more considered explicitly within the Born–Oppenheimer approximation. The modulus of each nuclear wavefunction is depicted for clarity.

We finally reach the final step of our pictorial derivation of the Born–Oppenheimer approximation. As stated above, the Born–Huang representation of the molecular wavefunction is formally exact and represents the molecular wavefunction as products ’electronic wavefunction $\times$ nuclear wavefunction’ summed over all electronic states. We discussed above that this representation allows us to accurately depict the dynamics of a molecule and the subtle couplings that exist between electronic and nuclear motion. We also observed that a molecule in a given electronic state (say, the ground electronic state) tends to remain in this electronic state whenever the nuclear motion is only weakly coupled to the electronic motion – for example, low nuclear momenta yielding $\tau _{\textrm{el}}/\tau _{\textrm{el}} \ll 1,$ or large energy separation between electronic states. In the Born–Huang picture, the latter statement appears as a nuclear wavefunction that keeps its amplitude in its electronic state throughout the process of interest (see Fig. 6), that is, a molecular wavefunction dominated by a *single* product ’electronic wavefunction $\times$ nuclear wavefunction’. It is therefore very tempting to approximate the total molecular wavefunction as such a single product ’electronic wavefunction $\times$ nuclear wavefunction’. This approximation would neglect the coupling between electronic and nuclear motions leading to transfer of nuclear amplitudes between electronic states – in other words, the molecule will always remain in a given electronic state independently of its nuclear motion. *This statement defines the Born–Oppenheimer approximation!* Figure 8 depicts a dynamics simulated within the Born–Oppenheimer approximation, employing the same initial condition as in Fig. 6 and resulting in a dynamics virtually identical to that presented in Fig. 6. Within the Born–Oppenheimer approximation, the molecular system is represented only by a nuclear wavefunction in the ground-electronic state, no other electronic states (or potential energy curves) are considered explicitly. The dynamics neglects all possible couplings between electronic and nuclear motion, which were in any case small in this example (see discussion about Fig. 6 above), leading to a good approximation of the nuclear (and overall molecular) dynamics. To quote Born and Huang, ’[...] during the nuclear motion, the electrons move as though the nuclei were fixed in their instantaneous positions. We say that the electrons follow the nuclear motion *adiabatically*. In an adiabatic motion, an electron does not make transitions from one state to others; instead, an electronic state itself is deformed progressively by the nuclear displacements. (Born and Huang 1954)

**Step 10: Final note: how does the total molecular wavefunction of our model system look like?**

As a final note, we would like to stress that the molecular wavefunction can always be recovered by multiplying the nuclear wavefunction by the electronic wavefunction, either for the unique electronic state of interest within the Born–Oppenheimer approximation or as a sum over all electronic states within the Born–Huang representation. The dimensionality of the molecular wavefunction makes it hard to represent for most molecular systems, but our simple models allows to depict it for the two limiting cases described above – an adiabatic case, where the molecule remains in its electronic ground-state throughout its dynamics, and a nonadiabatic case, where the molecule changes its electronic state due to a strong coupling between the nuclear and electronic motion (when the proton has a high momentum).

**Fig. 9:**
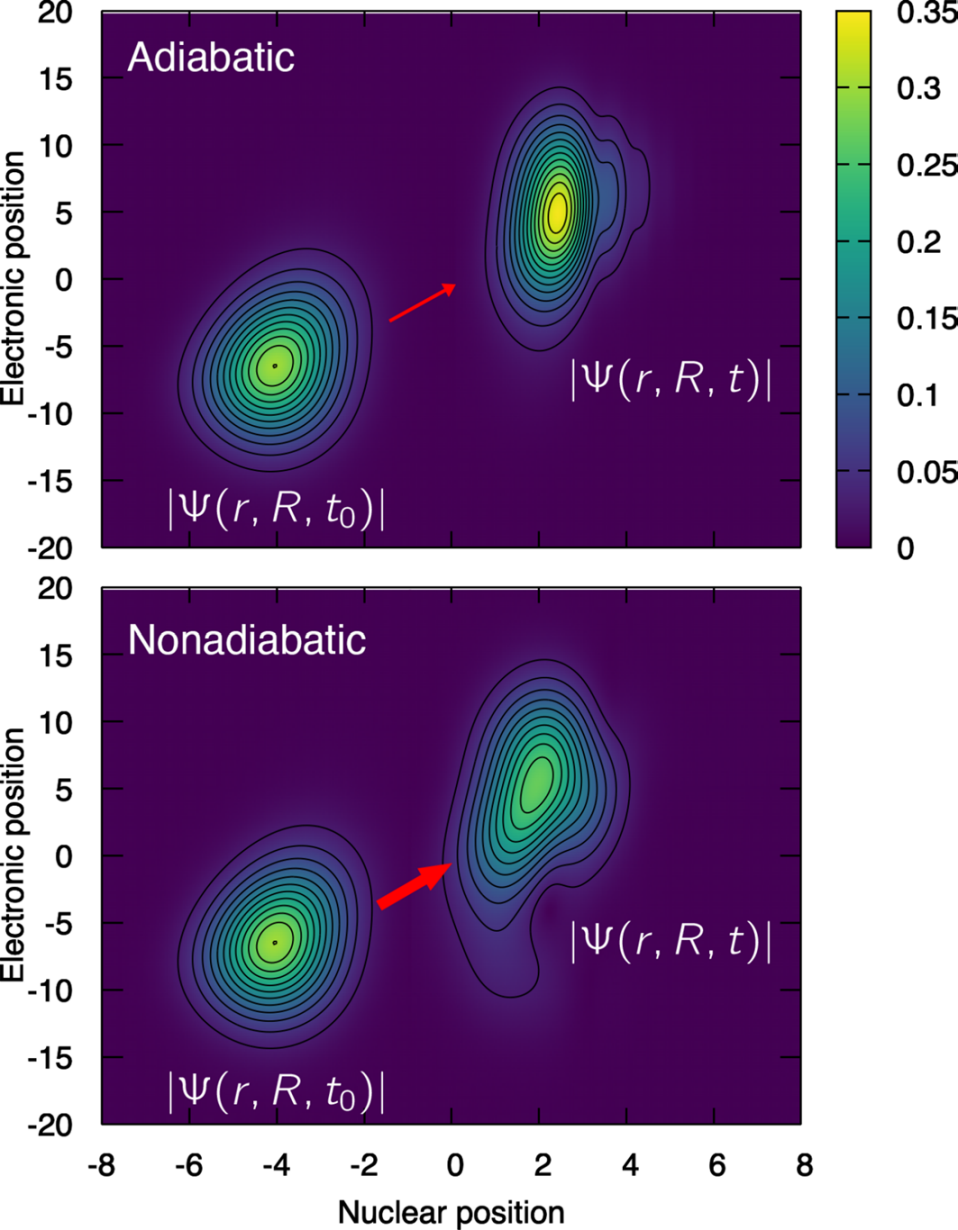
Evolution of the full molecular wavefunction from its initial state $\Psi (r,R,t_0)$ to a later time *t*, $\Psi (r,R,t)$ for the molecular model and for the two limiting cases discussed in this work: an adiabatic dynamics (upper panel) and a nonadiabatic dynamics (lower panel). The modulus of each molecular wavefunction is depicted for clarity.

Figure 9 depicts the evolution of the molecular wavefunction for the adiabatic (upper panel) and nonadiabatic (lower panel) cases. The abscissa represents the nuclear coordinate and the ordinate the electronic coordinate. In each plot, two snapshots are represented: the molecular wavefunction at the initial time $\Psi (r,R,t_0)$ and the molecular wavefunction at the final time $\Psi (r,R,t)$ studied above. In the adiabatic case, the molecular wavefunction depicts a dynamics where the electronic and nuclear coordinates follow each other closely (ripples can be observed at nuclear position larger than $R=3$, reminiscent to the plot of the nuclear wavefunction in Fig. 6). Such ’instantaneous’ relaxation of the fast electron around the motion of the slow nucleus is reminiscent to the general tenet of the Born–Oppenheimer approximation, and we discussed above that the Born–Huang representation justifies, for this specific case, the use of the Born–Oppenheimer approximation (i.e., the molecular wavefunction is well depicted by the dynamics of the nuclear wavefunction in a single electronic state). In the nonadiabatic case, the situation is different and the molecular wavefunction at time *t* appears more delocalized, spreading along the electronic coordinate, which highlights the coupling between electronic and nuclear motion leading to a growing importance of excited electronic states to depict the overall molecular dynamics (as observed in Fig. 7). The contribution of a component at around $r=-10$ to the molecular wavefunction is reminiscent to the electronic wavefunction for the second excited electronic state $\Phi _2(r;R)$ (see Fig. 4).

## Formal derivation of the Born–Oppenheimer approximation

In Sect. 2, we focused on a one-dimensional example depicted in Fig. 1 and will therefore use in the following the corresponding (simplified) formulation of the molecular time-dependent Schrödinger equation

1 $$\begin{aligned} i\hbar \frac{\partial }{\partial t} \Psi (r,R,t)=\hat{H}(r,R)\Psi (r,R,t) \, . \end{aligned}$$

The general form of the electrostatic potential is

2 $$\begin{aligned} \hat{V}_{\text {el}}(r,R)=\hat{V}_{\text {ee}}(r)+\hat{V}_{\text {nn}}(R)+\hat{V}_{\text {en}}(r,R) \, , \end{aligned}$$

but the one depicted in Fig. 2 does not include the electron–electron interaction $\hat{V}_{\text {ee}}(r)$ since the model contains only one electron. We use Eq. (2) to define the electronic Hamiltonian as

3 $$\begin{aligned} \hat{H}_{\text {el}}(r,R)=\hat{T}_{\text {e}}(r)+\hat{V}_{\text {el}}(r,R) \, . \end{aligned}$$

We stress that $\hat{V}_{\text {el}}(r,R)$ is a function of both the electronic and the nuclear coordinates. The molecular Hamiltonian of Eq. (1) has, then, the form

4 $$\begin{aligned} \hat{H}(r,R) = \frac{-\hbar ^2}{2M}\frac{d^2}{dR^2}+\hat{H}_{\text {el}}(r,R) \, , \end{aligned}$$

where the first term on the right-hand side is the nuclear kinetic energy operator expressed as a Laplacian, i.e., the second derivative with respect to the nuclear position.

The strategy introduced in Fig. 3 is achieved by discretizing the nuclear degree of freedom, namely $R \rightarrow R_1, R_2, R_3, \text {etc}$, defining the electrostatic potential as a function of the electronic coordinate *r* but for these specific nuclear coordinates, thus yielding a family of potentials as $\hat{V}_{\text {el}}(r,R_1), \hat{V}_{\text {el}}(r,R_2), \hat{V}_{\text {el}}(r,R_3), \text {etc}$. Adding now the kinetic energy operator for the electron, we obtain a discretized version – in nuclear space – of the electronic Hamiltonian, $\hat{H}_{\text {el}}(r,R_1), \hat{H}_{\text {el}}(r,R_2), \hat{H}_{\text {el}}(r,R_3), \text {etc}$. From this point on, we will label the discretized nuclear coordinates with a subscript *i* for simplicity.

We can now find, for a given nuclear configuration $R_i$, the eigenvalues and eigenvectors of the corresponding electronic Hamiltonian, namely, solving the electronic Schrödinger equation to exact electronic energies $(E^{\text {el}}_{J}(R_i))$ and electronic wavefunctions $(\Phi _J(r;R_i))$ for all electronic states:

5 $$\begin{aligned} \hat{H}_{\text {el}}(r,R_i) \Phi _J(r;R_i) = E^{\text {el}}_{J}(R_i)\Phi _J(r;R_i) \, . \end{aligned}$$

$\Phi _J(r;R_i)$ is the electronic wavefunction for electronic state *J*, which depends on the variable *r*, and the use of a semi-colon ’;’ indicates that this electronic wavefunction is defined by Eq. (5) for all nuclear configurations $R_i$. Figure 4 shows $|\Phi _J(r;R_i)|$ for $J=0,1,2$, from bottom to top, at the nuclear positions $R_i$. $E^{\text {el}}_{J}(R_i)$ is the corresponding adiabatic electronic energy for electronic state *J* at $R_i$. The adiabatic potential energy surfaces shown in Fig. 5 for $J=0,1,2$ are obtained by solving Eq. (5) for all values of $R_i$, and are thus functions of *R*.

Supposing that we can solve Eq. (5) for an infinitely fine grid in nuclear position *R*, we can abandon its discretize notation, and construct the Born–Huang representation of the molecular wavefunction as

6 $$\begin{aligned} \Psi (r,R,t)=\sum _J \Phi _J(r;R)\chi _J(R,t) \, . \end{aligned}$$

Since the electronic eigenfunctions $\Phi _J(r;R)$ are known for all values of the state index *J* and at all nuclear positions *R* from Eq. (5), the Born–Huang expansion (Eq. (6)) expresses the molecular wavefunction (still unknown) as a superposition, i.e., a sum, of electronic states, each contributing with an amplitude $\chi _J(R,t)$ (also unknown) to the sum. The nuclear wavefunctions are the projections of the molecular wavefunction onto the electronic eigenfunctions, i.e., $\chi _J(R,t)=\int \Phi _J^*(r;R) \Psi (r,R,t) dr$.

The Born–Huang representation given by Eq. (6) is simply a way to rewrite (exactly) the time-dependent molecular wavefunction, shifting the problem of determining the molecular wavefunction in time directly from the time-dependent Schrödinger equation to (i) an eigenvalue electronic problem, which is time independent as shown in Eq. (5), and (ii) a new time-dependent problem for all the nuclear wavefunctions contributing in Eq. (6). Step (1) is summarized by Figs. 4 and 5, while step (2) is depicted in Figs. 6 and 7 for the adiabatic and the nonadiabatic regimes, respectively.

The expressions yielding the time evolution of the nuclear wavefunctions for step (2) is obtained by inserting Eq. (6) into the time-dependent molecular Schrödinger equation (Eq. (1))

7 $$\begin{aligned} i\hbar \frac{\partial }{\partial t} \sum _J\Phi _J(r;R)\chi _J(R,t)=\left( \frac{-\hbar ^2}{2M} \frac{d^2}{dR^2}+\hat{H}_{\text {el}}(r,R)\right) \sum _J \Phi _J(r;R)\chi _J(R,t) \end{aligned}$$

and by calculating the action of the operators $\frac{\partial }{\partial t}$, $\frac{d^2}{dR^2}$ and $\hat{H}_{\text {el}}(r,R)$ on each term of the sum. The time derivative on the left-hand side of Eq. (7) only acts on $\chi _J(R,t)$ because $\Phi _J(r;R)$ does not depend on time, as it is the solution of the eigenvalue equation (5), thus

8 $$\begin{aligned} i\hbar \frac{\partial }{\partial t} \sum _J \Phi _J(r;R)\chi _J(R,t)=i\hbar \sum _J \Phi _J(r;R)\frac{\partial \chi _J(R,t)}{\partial t} \, . \end{aligned}$$

The Laplacian on the right-hand side of Eq. (7) acts on both $\Phi _J(r;R)$ and $\chi _J(R,t)$, since they both depend on *R*. Hence, the second-order derivative applied to the product of two functions that depend on *R* yields three terms,

9 $$\begin{aligned} \frac{-\hbar ^2}{2M} \frac{d^2}{dR^2}\sum _J \Phi _J(r;R)\chi _J(R,t) =&\frac{-\hbar ^2}{2M}\sum _J \chi _J(R,t)\frac{d^2\Phi _J(r;R)}{dR^2} \end{aligned}$$

10 $$\begin{aligned}&+\frac{-\hbar ^2}{2M}\sum _J 2 \frac{d\Phi _J(r;R)}{dR}\frac{d\chi _J(R,t)}{dR} \end{aligned}$$

11 $$\begin{aligned}&+\frac{-\hbar ^2}{2M}\sum _J \Phi _J(r;R)\frac{d^2\chi _J(R,t)}{dR^2}\, . \end{aligned}$$

Finally, the electronic Hamiltonian on the right-hand side of Eq. (7) acts on its eigenfunctions $\Phi _J(r;R)$ yielding the corresponding eigenvalues,

12 $$\begin{aligned} \hat{H}_{\text {el}}(r,R)\sum _J \Phi _J(r;R)\chi _J(R,t) = \sum _J E^{\text {el}}_{J}(R) \Phi _J(r;R)\chi _J(R,t)\, . \end{aligned}$$

We will now derive the evolution equations for all nuclear wavefunctions $\chi _J(R,t)$ of the Born–Huang expansion (7) in the following way. We left-multiply Eqs. (8) to (12) by $\Phi _K^*(r;R)$^3^ and we integrate over all possible electronic configurations *r*. Manipulating in this way Eq. (8), we obtain

13 $$\begin{aligned} \int \Phi _K^*(r;R)i\hbar \sum _J \Phi _J(r;R)\frac{\partial \chi _J(R,t)}{\partial t} dr&= i\hbar \sum _J \frac{\partial \chi _J(R,t)}{\partial t}\int \Phi _K^*(r;R)\Phi _J(r;R) dr \end{aligned}$$

14 $$\begin{aligned}&=i\hbar \frac{\partial \chi _K(R,t)}{\partial t} \, , \end{aligned}$$

where we used the orthonormality relation of the electronic eigenfunctions, i.e., $\int \Phi _K^*(r;R)\Phi _J(r;R) dr = \delta _{KJ}$. This relation means that if the (electronic) state indices are the same, i.e., $K=J$, then the scalar product $\int \Phi _K^*(r;R)\Phi _J(r;R) dr$ of the two eigenfunctions is 1 ($\delta _{KK}=\delta _{JJ}=1$), whereas if the indices are different, i.e., $K\ne J$, then the scalar product is 0 ($\delta _{KJ}=0$). The symbol $\delta _{KJ}$ is the Kronecker-$\delta$, which is equal to 1 if its indices are the same and zero otherwise. Note that the right-hand side of Eq. (13) is simply a rewriting of the left-hand side by taking out of the integral sign all quantities that do not depend on *r*, since the integral is performed on functions of *r* only. The final result of these operations is Eq. (14), featuring now a single *K* contribution rather than the sum over all electronic states we started with.

We repeat the same mathematical manipulations on Eqs. (9), (10) and (11). Therefore, we write

15 $$\begin{aligned} \frac{-\hbar ^2}{2M}\sum _J \chi _J(R,t) \int \Phi _K^*(r;R)\frac{d^2\Phi _J(r;R)}{dR^2} dr= \frac{-\hbar ^2}{2M}\sum _J \chi _J(R,t) D_{KJ}(R)\, , \end{aligned}$$

where we define the second-order nonadiabatic coupling as $D_{KJ}(R)=\int \Phi _K^*(r;R)\frac{d^2\Phi _J(r;R)}{dR^2} dr$,

16 $$\begin{aligned} \frac{-\hbar ^2}{2M}\sum _J 2\frac{d\chi _J(R,t)}{dR} \int \Phi _K^*(r;R)\frac{d\Phi _J(r;R)}{dR} dr = \frac{-\hbar ^2}{2M}\sum _J 2\frac{d\chi _J(R,t)}{dR} d_{KJ}(R) \, , \end{aligned}$$

where we define the (first-order) nonadiabatic coupling vector $d_{KJ}(R)=\int \Phi _K^*(r;R)\frac{d\Phi _J(r;R)}{dR} dr$, and

17 $$\begin{aligned} \frac{-\hbar ^2}{2M}\sum _J \frac{d^2\chi _J(R,t)}{dR^2} \int \Phi _K^*(r;R)\Phi _J(r;R)dr = \frac{-\hbar ^2}{2M}\frac{d^2\chi _K(R,t)}{dR^2} \, . \end{aligned}$$

Note that in our one-dimensional example, both $D_{KJ}(R)$ and $d_{KJ}(R)$ are scalar quantities, but in a more general situation only $D_{KJ}(R)$ is a scalar – $d_{KJ}(R)$ is a vector.

Applying the same manipulation to Eq (12) yields the final piece of the equations, namely

18 $$\begin{aligned} \sum _J E^{\text {el}}_{J}(R)\chi _J(R,t)\int \Phi _K^*(r;R)\Phi _J(r;R)dr = E^{\text {el}}_{K}(R)\chi _K(R,t)\, . \end{aligned}$$

Putting together Eqs. (14) to (18) and considering $K=0$, we can write the evolution equation for the $K=0$ nuclear wavefunction of the Born–Huang expansion of Eq. (7):

19 $$\begin{aligned} i\hbar \frac{\partial }{\partial t} \chi _0(R,t)&= \left( \frac{-\hbar ^2}{2M} \frac{d^2}{dR^2} + E^{\text {el}}_{0}(R)\right) \chi _0(R,t) + \sum _J \hat{C}_{0J}(R) \chi _J(R,t) \, . \end{aligned}$$

To avoid notation clutter, we collected the contributions from the first- and second-order nonadiabatic couplings into the expression $\hat{C}_{0J}(R)=\frac{-\hbar ^2}{2M}D_{0J}(R) -\frac{\hbar ^2}{M}d_{0J}(R)\frac{d}{dR}$. For $K=1, 2,3,\text {etc.}$ we obtain, similarly,

20 $$\begin{aligned} i\hbar \frac{\partial }{\partial t} \chi _1(R,t)&= \left( \frac{-\hbar ^2}{2M} \frac{d^2}{dR^2} + E^{\text {el}}_{1}(R)\right) \chi _1(R,t) + \sum _J \hat{C}_{1J}(R) \chi _J(R,t) \nonumber \\ i\hbar \frac{\partial }{\partial t} \chi _2(R,t)&= \left( \frac{-\hbar ^2}{2M} \frac{d^2}{dR^2} + E^{\text {el}}_{2}(R)\right) \chi _2(R,t) + \sum _J \hat{C}_{2J}(R) \chi _J(R,t) \nonumber \\ i\hbar \frac{\partial }{\partial t} \chi _3(R,t)&= \left( \frac{-\hbar ^2}{2M} \frac{d^2}{dR^2} + E^{\text {el}}_{3}(R)\right) \chi _3(R,t) + \sum _J \hat{C}_{3J}(R) \chi _J(R,t) \nonumber \\&\vdots \end{aligned}$$

Each one of these equations shows that the dynamics of the nuclear wavefunction *K*, which corresponds in the Born–Huang expansion to the contribution of the eigenfunction $\Phi _K(r;R)$ to the molecular wavefunction, is dictated by two terms: the first term is the sum of the nuclear kinetic energy operator $\frac{-\hbar ^2}{2M} \frac{d^2}{dR^2}$ and the electronic potential energy $E^{\text {el}}_{K}(R)$, whereas the second term is responsible for the coupling to all other nuclear wavefunctions *J* via the nonadiabatic coupling $\hat{C}_{KJ}(R)$. More specifically, this second term induces exchanges of nuclear amplitudes from one electronic state to another.

Figure 6 depicts an example of adiabatic dynamics, meaning that $\hat{C}_{0J}(R)\simeq 0$ for all values of the index *J* and at all *R*. Thus, if at the initial time the molecular wavefunction has non-zero contribution only in the ground state $K=0$, then the dynamics will remain in the ground state at all times *t* in the absence of nonadiabatic couplings to the other electronic states $J\ne 0$. As discussed in the Step 8 of Sect. 2, the evolution of the nuclei on the support of a single potential energy surface (in a single electronic state) yields an adiabatic dynamics,

21 $$\begin{aligned} i\hbar \frac{\partial }{\partial t} \chi _0(R,t)&= \left( \frac{-\hbar ^2}{2M} \frac{d^2}{dR^2} + E^{\text {el}}_{0}(R)\right) \chi _0(R,t) \, , \end{aligned}$$

since $$\hat{C}_{0J}(R)\simeq 0$$.

In the Born–Oppenheimer approximation, similarly to an adiabatic dynamics, the nuclear wavefunction with state index $K=0$ is constrained to evolve at all times on a single potential energy surface $\tilde{E}^{\text {el}}_{0}(R) = E^{\text {el}}_{0}(R) -\frac{\hbar ^2}{2M} D_{00}(R)$, where the effect of the so-called diagonal Born–Oppenheimer correction $D_{00}(R)$ is considered.^4^ Therefore, within the Born–Oppenheimer approximation, the molecular wavefunction is initiated in the ground state $K=0$ with zero contributions in all other electronic states and evolves in the electronic ground state under the effect of the potential energy $\tilde{E}^{\text {el}}_{0}(R)$, which describes the effect of the electrons,

22 $$\begin{aligned} i\hbar \frac{\partial }{\partial t} \chi _0(R,t)&= \left( \frac{-\hbar ^2}{2M} \frac{d^2}{dR^2} + \tilde{E}^{\text {el}}_{0}(R)\right) \chi _0(R,t) \, . \end{aligned}$$

Note that we considered here the explicit example of a ground-state dynamics, but the Born–Oppenheimer approximation can apply to dynamics in any other state. Also, in practical applications, the adiabatic Born–Oppenheimer approximation is often used, namely Eq. (22) without considering the effect of the diagonal Born–Oppenheimer correction.

Finally, it is important to stress that Eq. (22) is consistent with the Ansatz

23 $$\begin{aligned} \Psi (r,R,t) \simeq \Psi _{\text {BOA}}(r,R,t) = \Phi _K(r;R)\chi _K(R,t) \end{aligned}$$

which is an alternative way of formulating the Born–Oppenheimer approximation by directly using an approximation to the molecular wavefunction, limiting its definition to a single term of the full Born–Huang expansion.

## Summary and final considerations

We presented a pictorial derivation of the Born–Oppenheimer approximation and showed that this key approximation in chemistry does not imply that the nuclei of a molecule are frozen, nor that they are classical. We showed that these misconceptions often emerge from a confusion in the mathematical steps necessary to reach the Born–Oppenheimer approximation, as summarized below for clarity.

**Confusion 1: The Born–Oppenheimer approximation does not mean that we set the kinetic energy operators for the nuclei to zero in the molecular Hamiltonian** The Born–Oppenheimer approximation is very often confused with the action of setting the kinetic energy for the nuclei to zero in the molecular Hamiltonian. As discussed above, this statement is clearly wrong and emanates from the very first step of the Born–Huang representation, which relies on the definition of the basis of electronic wavefunctions to express the molecular wavefunction. In this first step, we focus on determining the electronic eigenvalues and eigenfunctions for any possible nuclear displacement, requiring the use of the electronic Hamiltonian, which is the same as the molecular Hamiltonian with the kinetic energy operator for the nuclei set to zero. The confusion between this step of the Born–Huang derivation and the Born–Oppenheimer approximation (where only specific contributions of the kinetic energy operator for the nuclei – the nonadiabatic coupling terms – are neglected) may have been further reinforced by the notion that the Born–Oppenheimer approximation involves ’frozen nuclei’ (zero kinetic energy for the nuclei), which is also incorrect as discussed in this article. If nuclei were to be frozen within the Born–Oppenheimer approximation, there would be no chemistry under this approximation! Setting to zero the nuclear kinetic energy can sometimes be associated wrongly to the operation $M\rightarrow \infty$ derived from the Born–Oppenheimer approximation formulated as $m/M \rightarrow 0$, thus we recommend extreme care in taking this limit and to follow previous work in this direction (Hagedorn 1986, Eich and Agostini 2016).

**Confusion 2: The Born–Oppenheimer approximation does not lead to a classical description of the nuclear degrees of freedom** An important aspect of the derivation of the Born–Oppenheimer approximation is that, at no point, the nuclear degrees of freedom are required to become classical. The nuclei remain quantum within the Born–Oppenheimer approximation: we are still describing the state of the nuclei by a (single) nuclear wavefunction. In other words, the molecular wavefunction, within the Born–Oppenheimer approximation, is described by a single ’electronic wavefunction $\times$ nuclear wavefunction’ product, often taken for the ground-electronic state. Note that this single product involves a nuclear wavefunction, i.e., a quantum description of the nuclear degrees of freedom. Hence, the Born–Oppenheimer approximation does not mean that we describe the nuclei classically. It is possible, though, to take a classical limit on the nuclei following the Born–Oppenheimer approximation (see, for example, Marx and Hutter 2009) but this comes as an additional approximation.

We finally would like to stress two important considerations for chemistry emanating from the Born–Oppenheimer approximation.

**Consideration 1** Born–Huang and Born–Oppenheimer bring the picture of chemistry we love and use: nuclei that stick together thanks to the electrons – the chemical bonds – or, said in a different way, the nuclei evolve in a potential created by the electrons. All pictures of chemistry, e.g., chemical reaction pathway on a potential energy surface, photochemistry with different electronic states, emerge from the Born–Huang representation of the molecular wavefunction. In the abstract of Tully 2000, John C. Tully says that *The Born–Oppenheimer approximation, introduced in the 1927 paper “On the quantum theory of molecules”, provides the foundation for virtually all subsequent theoretical and computational studies of chemical binding and reactivity, as well as the justification for the universal “ball and stick” picture of molecules as atomic centers attached at fixed distances by electronic glue.* Eric J. Heller, in his book (Heller 2018), states that *Without the Born–Oppenheimer approximation, there would be no molecular structure, no solid-state crystal structure, no molecular vibrations, no phonons, no electronic band structure, and so on. Why? Because it is the Born–Oppenheimer approximation that allows separations of electronic from nuclear motion. Without it, we appear to be lost in a soggy many-body “pea soup” or plasma of electrons and nuclei, where there is seemingly no structure at all, save the kind of structure one finds in a two-component liquid*. Finding approximations to the electronic Schrödinger equation is a research field often coined ’electronic-structure theory’, or when focusing on molecular systems ’quantum chemistry’. We also note that various efforts – pre-Born–Oppenheimer theory (Matyus 2019) and exact factorization (Abedi et al. 2010 and Abedi et al. 2012) – have emerged over the recent years to make sense of the many-body soup without relying on the Born–Oppenheimer approximation or Born–Oppenheimer states.

**Consideration 2** Quantum chemists often locate specific molecular structures – an optimized ground-state geometry or a transition-state structure – to characterize a chemical reaction pathway. While this strategy may appear as ’fixing the nuclei to a given, local geometry’, it is key to realize that these chemical structures are not believed to represent the molecular system in themselves, but are often taken as a starting point for further calculations. For example, using the harmonic approximation to describe the local potential energy surface around a located critical geometry allows to approximate the ground-state vibrational wavefunction for the system (and correction to the electronic energy to account for the quantum nature of the nuclei). It is nevertheless possible to look at electronic-structure quantities (e.g., molecular orbitals for molecules or band structures for solids or materials) at a fixed nuclear position (for example, an optimized geometry), yet one should realize that this approach implies an additional approximation on top of the Born–Oppenheimer approximation: the nuclei are considered as fixed (classical) point charges.

## Data Availability

No datasets were generated or analysed during the current study.

## Footnotes

1 We note that we could have equally discretized the total electrostatic potential along the electronic positions. This strategy was employed for specific cases, see for example Suzuki et al. (2014).

2 We should stress at this point that we discuss here the time-dependent molecular Schrödinger equation – interested in the time evolution of the molecular wavefunction – but the Born–Huang representation applies as well to the time-independent molecular Schrödinger equation – focusing then on the determination of the possible stationary molecular eigenstates for the molecule. (Tully 2000)

3 Note that, since the eigenfunctions of the electronic Hamiltonian are, in general, complex-valued functions, the symbol $^*$ indicates the operation of taking the complex conjugate.

4 Note that it can be shown that the diagonal contributions of the first-order nonadiabatic couplings $d_{KK}(R)$ are 0 for all value of *K* if the electronic wavefunctions are real, thus they are not considered in the diagonal Born–Oppenheimer correction.
